# Longitudinal Brain Development of Numerical Skills in Typically Developing Children and Children with Developmental Dyscalculia

**DOI:** 10.3389/fnhum.2017.00629

**Published:** 2018-01-04

**Authors:** Ursina McCaskey, Michael von Aster, Urs Maurer, Ernst Martin, Ruth O'Gorman Tuura, Karin Kucian

**Affiliations:** ^1^Center for MR-Research, University Children's Hospital Zurich, Zurich, Switzerland; ^2^Children's Research Center, University Children's Hospital Zurich, Zurich, Switzerland; ^3^Clinic for Child and Adolescent Psychiatry, German Red Cross Hospitals, Berlin, Germany; ^4^Neuroscience Center Zurich, University of Zurich and Swiss Federal Institute of Technology Zurich, Zurich, Switzerland; ^5^Department of Psychology, University of Zurich, Zurich, Switzerland; ^6^Department of Psychology, Chinese University of Hong Kong, Hong Kong, Hong Kong; ^7^Zurich Center for Integrative Human Physiology, University of Zurich, Zurich, Switzerland

**Keywords:** brain development, child, developmental dyscalculia, longitudinal, number processing

## Abstract

Developmental dyscalculia (DD) is a learning disability affecting the acquisition of numerical-arithmetical skills. Studies report persistent deficits in number processing and aberrant functional activation of the fronto-parietal numerical network in DD. However, the neural development of numerical abilities has been scarcely investigated. The present paper provides a first attempt to investigate behavioral and neural trajectories of numerical abilities longitudinally in typically developing (TD) and DD children. During a study period of 4 years, 28 children (8–11 years) were evaluated twice by means of neuropsychological tests and a numerical order fMRI paradigm. Over time, TD children improved in numerical abilities and showed a consistent and well-developed fronto-parietal network. In contrast, DD children revealed persistent deficits in number processing and arithmetic. Brain imaging results of the DD group showed an age-related activation increase in parietal regions (intraparietal sulcus), pointing to a delayed development of number processing areas. Besides, an activation increase in frontal areas was observed over time, indicating the use of compensatory mechanisms. In conclusion, results suggest a continuation in neural development of number representation in DD, whereas the neural network for simple ordinal number estimation seems to be stable or show only subtle changes in TD children over time.

## Introduction

How does the “numerical brain” develop? Numbers are omnipresent in our lives and our innate ability to detect small numerosities enables us to develop complex mathematical skills at a young age (Starkey et al., 1990; Xu and Spelke, 2000; Izard et al., 2009). Not surprisingly, individuals with Developmental Dyscalculia (DD) struggle in their everyday life. DD is a learning disability affecting the acquisition of numerical-arithmetical skills in children with normal intelligence and age-appropriate school education (WHO, 2010). Many studies have shown that children with DD display various deficits in number processing skills such as magnitude processing or spatial number representation (Landerl et al., 2004; Rousselle and Noël, 2007; Mussolin et al., 2010; Landerl, 2013). Those skills are assumed to predict later arithmetical achievement (Halberda et al., 2008; De Smedt et al., 2009; Geary et al., 2012; Träff, 2013) and are therefore essential for the development of numeracy. DD has a high prevalence (3–7%) (Gross-Tsur et al., 1996; Wyschkon et al., 2009; Reigosa-Crespo et al., 2012) and a persisting character (Shalev et al., 1998, 2005). The fact that difficulties in numeracy result in reduced employment opportunities and high public costs underscores the importance of understanding more about numerical brain development (Parsons and Bynner, 2005; Gross, 2009).

Research performed over the last decades demonstrates that from the first day after birth, infants are capable of discriminating quantities (Xu et al., 2005; Izard et al., 2009) and show specialized neuronal correlates for the processing of numerosities early in development (Hyde et al., 2010; Hyde and Spelke, 2012). Over development, a spatial representation of quantity and numbers, also known as mental number line (Berch et al., 1999; Dehaene, 2003), emerges. With the acquisition of number words and the symbolic number system, the formation of such an internal representation further refines (Siegler and Booth, 2004; von Aster and Shalev, 2007; Ebersbach et al., 2008; Halberda and Feigenson, 2008). Moreover, numerical magnitude processing skills (linearity of the mental number line, performance in quantity comparison tasks) correlate with arithmetical knowledge and predict future mathematical achievement (Booth and Siegler, 2008; De Smedt et al., 2013). A recent study further showed that number line estimation is a good predictor of arithmetic ability at an early age, whilst ordinal processing of numerical symbols was revealed to be a strong predictor of older children's arithmetical skills (Lyons et al., 2014; Zhu et al., 2017). Besides various other deficits in numerical-arithmetical skills, several studies with DD children reported that they are less accurate in placing numbers on a number line (Geary et al., 2008; Landerl, 2013). Piazza et al. (2010) showed that DD children performed at a similar level as 5-years-younger typically developing (TD) children in a task measuring number representation. Furthermore, results of a review reveal that weak performance of magnitude processing skills correlates with low mathematical achievement and DD (De Smedt et al., 2013). These results are supported by neuroimaging findings demonstrating that children with DD show aberrant functional activation in number tasks compared to TD peers. Significantly reduced activation is mainly found in domain-specific regions of the parietal lobe, known to be important for magnitude and ordinal processing and supposed to incorporate the mental number line (Kucian et al., 2006, 2011a; Price et al., 2007; Mussolin et al., 2010; Ashkenazi et al., 2012). For instance, children with DD showed reduced activation in the bilateral intraparietal sulcus (IPS) and superior parietal lobe when solving a number processing task (Kucian et al., 2011a). Moreover, when confronted with arithmetical problems, DD children failed to show a task related modulation in parietal areas. However, findings are not consistent and some studies describe increased activation in DD in these areas (Davis et al., 2009; Kaufmann et al., 2009b, 2011). Rosenberg-Lee et al. (2015), for instance, reported that children with DD show hyper-activation in parietal cortices when solving subtraction problems. Moreover, activation differences are also found in domain-general regions mainly in the frontal brain, attributed to working memory, attention and planning, but also in occipito-temporal areas of the brain (Kucian et al., 2006, 2011a; Price et al., 2007; Davis et al., 2009; Kaufmann et al., 2009b; Rosenberg-Lee et al., 2015). Recent studies further revealed that children with DD show functional hyper-connectivity of the IPS with the bilateral fronto-parietal network (Jolles et al., 2016; Michels et al., 2017). To summarize, these findings describing an aberrant brain activation pattern possibly reflect the typical deficiency in number processing and the greater cognitive resources needed to solve numerical tasks.

A number of cross-sectional studies have been conducted to investigate age dependent neural differences of numerical functions in TD children. Findings suggest that children activate similar regions to adults when solving numerical tasks (Peters and De Smedt, in press). However, children recruit parietal regions to a lesser extent, in particular the IPS, and show increased frontal activation compared to adults (Ansari et al., 2005; Ansari and Dhital, 2006; Cantlon et al., 2006; Kucian et al., 2008; Holloway and Ansari, 2010). According to these findings, researchers hypothesized that there is a shift from an initially controlled and effortful (frontal activation) to a subsequently more automatic processing of numerical magnitude (parietal activation) (Ansari et al., 2005; Rivera et al., 2005; Kucian et al., 2008; Holloway and Ansari, 2010). Conversely, Rosenberg-Lee et al. (2011) reported an increase in parietal, but also prefrontal and visuo-temporal regions over 1 year in children solving arithmetic problems, suggesting a nonlinear trajectory of development.

Several behavioral long-term studies investigated the development of typical and atypical number processing (such as dot enumeration, counting, and number comparison), showing that its efficiency is a good predictor for arithmetical achievement (Halberda et al., 2008; Desoete et al., 2012; Geary et al., 2012; Passolunghi and Lanfranchi, 2012; Landerl, 2013; Reigosa-Crespo et al., 2013; Träff, 2013). Landerl (2013) followed children's numerical abilities over 2 years and found that even if dyscalculic children showed improvements, numerical processing remains persistently deficient. This is also in line with the results of a systematic review about longitudinal studies of mathematical difficulties indicating that students with math difficulty improve in mathematical measures over time but do not catch up to their peers (Nelson and Powell, 2017). Further studies revealed that those deficits are already detectable in kindergarten and continue to persist into adolescence (Shalev et al., 1998, 2005; Stock et al., 2009; Geary et al., 2013; Mazzocco et al., 2013).

To date, the current body of research has identified a substantial deficit in numerical processing in children with DD. Studies with TD subjects indicate that there is a functional specialization in the areas devoted to numerical magnitude representation and involved in the development of the mental number line. On the neural level, DD is associated with aberrant activation patterns of the number-specific parietal regions and domain-general areas. Nevertheless, little is known about the neural development of numerical abilities.

Hence, the goal of the present study was to investigate the typical and atypical neural development of numerical abilities by means of longitudinal functional Magnetic Resonance Imaging (fMRI) and behavioral data. With fMRI we investigated the ordinal aspect of number processing, as differences between DD and TD children have been reported in parietal and domain general regions during a numerical order task (Kucian et al., 2011a). In addition, ordinal number processing has been shown to be an important predictor for arithmetic skills as development progresses (Lyons et al., 2014). Together with behavioral measures on the spatial representation of quantity and numbers (number line task) we aimed to provide insight into the development of numerical abilities.

Evidence from studies with TD children and adults revealed a shift from frontal to parietal activation over time. Based on this literature, we expect to find an increase in activation in the number-specific parietal regions and a decrease in the domain-general regions reflecting the growing proficiency in number processing in TD children (Ansari and Dhital, 2006; Cantlon et al., 2006; Holloway and Ansari, 2010). To our knowledge there are no studies about the neuro-functional development of children with DD, making predictions about the atypical development difficult. However, studies show that children and adults with DD show aberrant activation in the number-specific parietal areas (Molko et al., 2003; Kucian et al., 2006, 2011a; Kaufmann et al., 2009a). Furthermore, longitudinal behavioral findings show that children with DD show persistent deficits in numerical processing (Geary et al., 2013; Landerl, 2013; Nelson and Powell, 2017). In line with these findings, we hypothesize a persistent deficiency in numerical processing and consistently lower parietal activity in children with DD compared to TD children (Kucian et al., 2006, 2011a; Price et al., 2007). As we predict persistent aberrant parietal activity, but at the same time improvements in number processing (Landerl, 2013; Nelson and Powell, 2017), we further expect to find higher frontal activation over time in DD children reflecting the changes in the requirement of the cognitive resources as a result of a delayed development.

## Materials and methods

### Study design and participants

**Figure 1:**
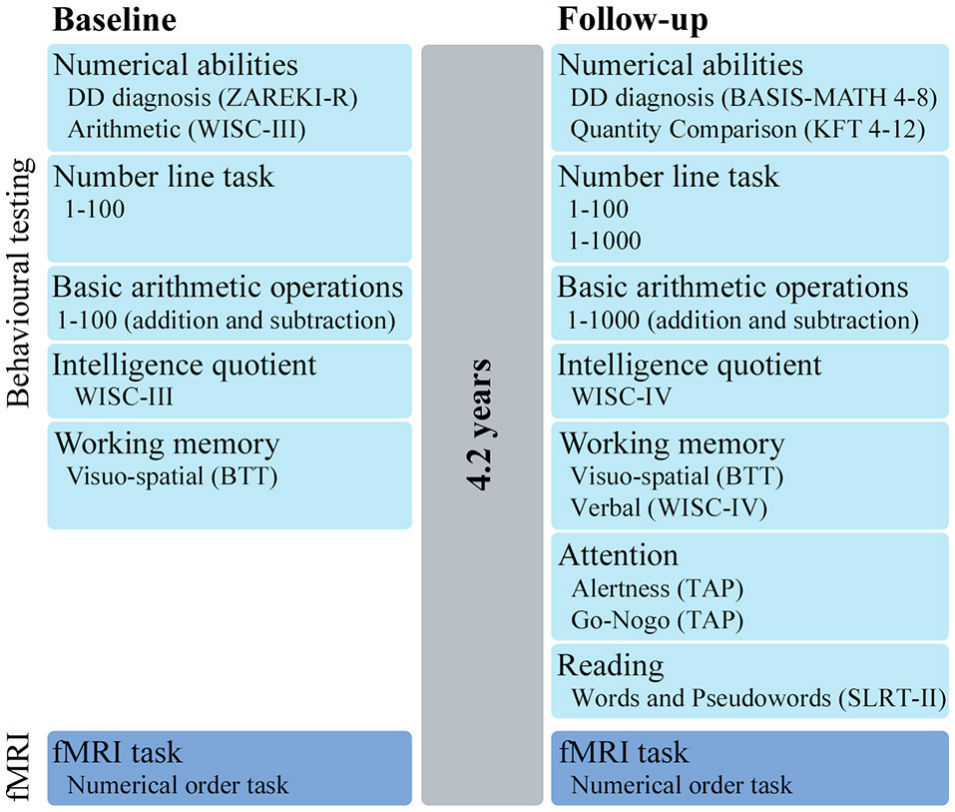
Study design. Overview of the behavioral tests and fMRI task performed at baseline and after 4.2 years at follow-up. ZAREKI-R, Neuropsychological Test Battery for Number Processing and Calculation in Children; BASIS-MATH 4–8, Basic Diagnostic in Mathematics for Grades 4–8; KFT 4–12, Cognitive Abilities Test; WISC, Wechsler Intelligence Scale for Children; BTT, Block-Tapping-Test; TAP, Testbattery for Attentional Performance; SLRT-II, Salzburg Reading and Orthography Test.

In this longitudinal study, a group of children with DD and a group of TD children were evaluated by neuropsychological tests and fMRI (baseline). After 4.2 (SD = 0.46) years, children returned for a second neuropsychological and fMRI assessment (follow-up) (Figure 1).

In total 35 (23 DD, 12 TD) children between 8 and 11 years were recruited into this study, of which 25 took part in a previous study (Kucian et al., 2011a) (Note that while the previous study acquired data both before and after number line training, only the pre-training data from this previous study were included as a baseline measurement for the present study. Therefore, all children included in the present study participated for the first time in a study at our MRI center and performed the same behavioral tests and fMRI paradigm). Inclusion criteria for all children were an IQ > 85 and no history of a neurologic or psychiatric disorder. Additionally, DD children had to perform below the 10th percentile in the total score or three subtests of a standardized numerical test battery (ZAREKI-R) at baseline. TD children required age-appropriate mathematical performance at baseline and follow-up, defined as performing above the 10th percentile in the ZAREKI-R (range of the TD children: PR 46-100) and above the cut-off of 67 points in the BASIS-MATH 4-8 (range of the TD children: 68–83) (see also Figure 1 and Supplementary Material). According to these criteria, six DD children were excluded because they exceeded the cut-off in the numerical test and one TD child because of medication. Therefore, the behavioral data analyses are based on 17 DD and 11 TD children.

For the fMRI analysis, three data sets at baseline and seven at follow-up were excluded because of task performance < 50% (1 data set), scanner problems (3 data sets) or poor image quality caused by dental braces (6 data sets). Hence, subsequent statistical group comparisons are based on 14 DD and 11 TD fMRI data sets at baseline, and 13 DD and 8 TD fMRI data sets at follow-up.

Informed and written consent was obtained from participants when older than 16 years and all parents. The study was approved by the Ethics committee of Zurich, Switzerland based on guidelines from the World Medical Association's Declaration of Helsinki (WMA, 2002).

### Behavioral testing

All children completed age-appropriated neuropsychological tests at baseline and follow-up (for an overview see Figure 1).

#### Handedness

Handedness (3 left handed, 8 ambidextrous, 17 right handed) was determined by the Edinburgh Handedness Inventory (Oldfield, 1971).

#### Diagnosis of DD and general numerical abilities

At baseline, numerical abilities were assessed using the revised version of the Neuropsychological Test Battery for Number Processing and Calculation in Children (ZAREKI-R) (von Aster et al., 2006). This test battery consists of 12 subtests assessing basic numerical skills as well as calculation (see Supplementary Material for detailed information about the subtests). Based on this test battery children with DD were identified from scores below the 10th percentile in three subtests or in the total test score (test scores are reported in percentile ranks). At the follow-up assessment, the test for Basic Diagnosis in Mathematics Education for Grades 4–8 (BASIS-MATH 4–8) (Moser Opitz et al., 2010) was used instead, because it is the only German test in existence which can identify numerical deficiencies up to the eighth grade. The BASIS-MATH test battery is composed of three difficulty levels measuring several arithmetical abilities (see also Supplementary Material). The test battery assumes that mastery of basic mathematical concepts is not reached, if the performance falls under a threshold value of 67 points (out of total 83 points, reported test scores are raw values).

In order to asses children's arithmetic performance at the peer level, the Arithmetic subtest of the Wechsler Intelligence Scale for Children (WISC-III) (Tewes et al., 1999) was performed at baseline. In this subtest children had to solve story problems of increasing difficulty within a set time limit (reported test values are IQ scores). At the follow-up measurement, the Quantity Comparison subtest of the Cognitive Abilities Test (KFT 4-12+R) (Heller and Perleth, 2000) was performed. In the Quantity Comparison subtest subjects had 10 min time to solve as many quantity comparisons as possible of increasing difficulty (reported test values are T scores).

#### Number line task

The spatial representation of numbers was measured by means of a paper-and-pencil number line task adopted from Kucian et al. (2011a). Children had to estimate the position of 20 Arabic digits on a left to right oriented number line (length 16 cm) with the labeled end points 0 and 100. A single number was presented verbally as well as visually in form of an Arabic digit on a card. Each number had to be marked on consecutive number lines to avoid the possibility of comparisons between items. Two items per decade were chosen in order to evaluate the entire spatial representation between 0 and 100.

At the follow-up, a computerized and age-adapted version of the number line task was used. Each of the 20 numbers was presented visually on the screen and its position was indicated by mouse-click. The number line was 21.3 cm in length (806 pixel, with a screen resolution 96 dpi) and had labeled end points. For the number range 0–100, two numbers per decade were selected again. Additionally, participants had to solve 20 items in the number range between 0 and 1000. To obtain the items in the range 0–1000, the numbers of the number line test 0–100 were multiplied by 10 and a random digit between 0 and 9 was added in the unit position.

In both test versions accuracy was measured by calculating the percentage distance from the marked to the correct position of the given number (reported test values are raw values).

#### Basic arithmetic operations

Children solved 40 basic arithmetic problems (20 addition and 20 subtraction) (Kucian et al., 2011a). Each problem was presented verbally as well as visually on a card. The child had to provide the solution verbally and the examiner noted it on the evaluation sheet. There was no time limit for this test. The items ranged from 1 to 100 with single digit as well as double digit problems (e.g., 7+15, 36+42). The items were balanced for frequency of digits and bridging ten. The number of correctly solved items was quantified (reported test values are raw scores, maximum value 20).

At the follow-up, a computerized and age-adapted version of this task was used. Each of the 20 addition and 20 subtraction was presented visually on the screen and solutions were typed on a keyboard. There was no time limit for this test. To prevent ceiling effects, the test was expanded to numbers up to 1000. Items consisted of one-, two- and three-digit numbers (e.g., 811+5, 235+324) and were balanced for frequency of digits and bridging ten/hundred. RT was measured and the number of correctly solved items was quantified (reported test values are raw scores, maximum value 20).

#### Intelligence quotient

Intelligence was measured with the third, respectively fourth edition of the WISC (Tewes et al., 1999; Petermann and Petermann, 2007) (WISC-III: Similarities, Block Design, Vocabulary, Picture Arrangement; WISC-IV: Similarities, Block Design, Matrix Reasoning). Table 1 shows the estimated general IQ (reported test values are IQ scores).

**Table 1:** Demographic characteristics and scores on numerical abilities, intelligence quotient, working memory, attention, and reading.

**Behavioral measure**	**DD**		**TD**		**Test- statistic**	***p***
	**N**	***M (SD)***	**N**	***M (SD)***		
**BASELINE ASSESSMENT**						
Age	17	9.6 (0.8)	11	9.1 (0.9)	1.51^a^	0.144
Gender m/f	17	3/14	11	6/5	4.17^b^	0.095
Handedness l/a/r	17	2/5/10	11	1/3/7	0.08^b^	0.999
Numerical abilities						
DD diagnosis *(ZAREKI-R)*	17	6 (4.9)	11	77 (19.1)	−12.12^a^	< 0.001^***^
Arithmetic *(WISC-III)*	16	92 (8.7)	11	107 (13.5)	−3.34^a^	0.004^**^
Estimated IQ *(WISC-III)*	17	100 (6.4)	11	112 (6.9)	−4.65^a^	< 0.001^***^
Working memory						
Visuo-spatial *(BTT)*	14	2.9 (1.8)	11	3.7 (1.0)	−1.44^a^	0.164
**FOLLOW-UP ASSESSMENT**						
Age	17	13.8 (1.0)	11	13.5 (0.9)	0.80^a^	0.429
Numerical abilities						
DD diagnosis	17	52.2 (9.4)	11	76.0 (4.6)	−7.80^a^	< 0.001^***^
*(BASIS-MATH 4–8)*						
Quantity Comparison	13	41.3 (3.4)	11	54.6 (5.9)	−6.90^a^	< 0.001^***^
*(KFT 4-12+R)*						
Estimated IQ *(WISC-IV)*	17	102 (7.3)	11	113 (5.4)	−4.41^a^	< 0.001^***^
Working memory						
Visuo-spatial *(BTT)*	16	5.6 (1.7)	11	7.0 (2.0)	1.16^c^	0.039^*^
Verbal *(*WISC*-IV)*	17	4.4 (1.0)	11	5.0 (1.0)	−1.68^a^	0.106
Attention *(TAP)*						
Alertness	16	46 (10.5)	11	47 (9.9)	−0.07^a^	0.946
Go-Nogo	16	63 (31.2)	11	66 (24.5)	−0.28^a^	0.780
Reading *(SLRT-II)*						
Words	16	37 (26.7)	11	42 (27.0)	12.28^b^	0.765
Pseudowords	14	42 (25.6)	11	46 (23.1)	11.54^b^	0.619

*ZAREKI-R, Neuropsychological Test Battery for Number Processing and Calculation in Children [PR], BASIS-MATH 4–8, Basic Diagnostic in Mathematics for Grades 4–8 [raw score]; WISC, Wechsler Intelligence Scale for Children [IQ score]; KFT 4-12+R, Cognitive Abilities Test [T score]; BTT, Block-Tapping-Test [raw score]; TAP, Testbattery for Attentional Performance [PR]; SLRT-II, Salzburg Reading and Orthography Test [PR]*.

a *t-Test*.

b *Fisher's Exact Test*.

c *Kolmogorov-Smirnov-Z Test*.

* *p < 0.05*,

** *p < 0.01*,

*** *p < 0.001*.

#### Working memory

Visuo-spatial and verbal working memory was assessed in order to control for memory effects. At baseline and follow-up working memory was measured with the Block-Suppression-Test (Beblo et al., 2004). The task required subjects to reproduce every second block of a previous presented sequence on a board with nine cubes. The sequences had a length of 3–9 cubes. Three items per sequence were presented. The longest sequence which was reproduced correctly twice was quantified (reported test values are raw scores, maximum value 9).

At the follow-up the subtest Digit Span of the WISC-IV (Petermann and Petermann, 2007) was additionally performed. In this task subjects had to repeat an auditorily presented sequence of numerals backwards. The sequences had a length of 2 to 8 numerals. The longest sequence which was reproduced correctly was quantified (reported test values are raw scores, maximum value 8).

#### Attention

Levels of attention and inhibition were measured at follow-up by means of the subtests Alertness and Go-Nogo of the computerized Testbattery for Attentional Performance (TAP) (Zimmermann and Fimm, 1993). In the Alertness subtest, subjects had to react as quickly as possible when the target stimulus “X” appeared (intrinsic alertness). Half of the trials were preceded by an acoustic cue stimulus (phasic alertness). The test has four runs and a total of 80 target items. For each subject the percentile rank of the median RT was quantified (reported test values are percentile ranks). In the Go-Nogo subtest, subjects had to react as quickly as possible to a target stimulus (“X,” go condition), but inhibit reactions on a second presented stimulus (“+,” nogo condition). The test has a total of 40 items (20 go and 20 nogo items). For each subject the percentile rank of the median RT was quantified (reported test values are percentile ranks).

#### Reading

The 1-Min-Reading-Task from the Salzburg Reading and Orthography Test (SLRT-II) (Moll and Landerl, 2010) assessing word and pseudoword reading fluency was used to estimate the reading performance at follow-up. Two sheets of paper with either 156 words or 156 pseudowords of increasing length and difficulty were presented. Subjects had 1 min per sheet to read as many words as possible. The amount of correctly read items was quantified (reported test values are percentile ranks). Because of lacking test norms in grades 7 and 8, we interpolated the norms from the test manual (grade 6) and from Kronschnabel et al. (2013) (grade 9).

### fMRI task

**Figure 2:**
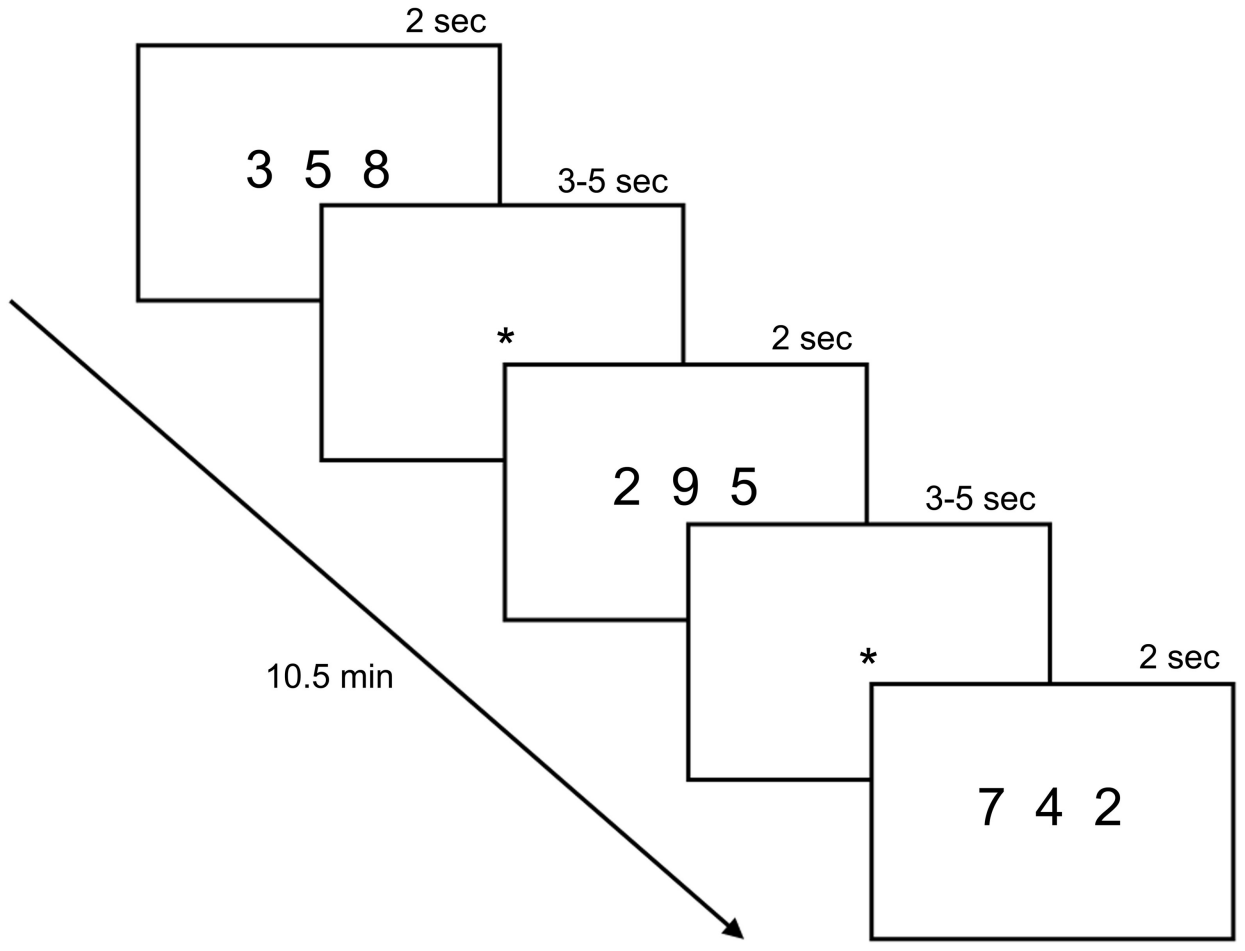
fMRI task. The paradigm consisted of alternating blocks of the experimental and control condition. In the experimental condition, subjects had to decide if the three presented numbers were in ascending or descending order (numerical order task). For instance, subjects had to press “yes” in the first and last trial shown in the picture and “no” for the second one. In the control condition, subjects had to indicate if the number 2 was present. Every stimulus was presented for 2 s, followed by a screen with a fixation cross (^*^) shown for 3–5 s. Reprinted from Kucian et al. (2011a), copyright (2011) with permission from Elsevier.

The fMRI task, adopted from Kucian et al. (2011a), was identical between the baseline and follow-up measurements. In the experimental condition, subjects had to make ordinal judgements (numerical order task: “Are the numbers in an ascending/descending order?”). The control condition was a number identification task (“Is the number 2 present?”) (Figure 2). The entire paradigm lasted 10.5 min and consisted of four blocks of the numerical order task alternating with four blocks of the number identification task. Blocks were counter-balanced between subjects. At the beginning of each block an instruction was shown for 2 s, followed by 10 trials of one of the two conditions and a rest period with a fixation cross for 20 s, resulting in a total block length of 59.5 s. Every stimulus was presented for 2 s, followed by a blank screen with an inter-stimulus-interval jittered between 3 and 5 s.

A stimulus consisted of three Arabic digits between “2” and “9” (horizontally aligned) shown simultaneously via a video goggles system (VisuaStimDigital, Resonance Technology Inc., USA). 40 numerical order items were presented, one fourth with ascending (correct), one fourth with descending (correct) and half of them with no specific order (incorrect). The order of the numerals in the ascending condition (e.g., 2 5 7) was reversed to obtain the descending items (e.g., 7 5 2) and mixed up to obtain the items with no specific order (e.g., 5 2 7). In the control condition, 40 number identification items were presented (20 correct and 20 incorrect items). The paradigm was balanced for numerical distance [max(*n*)-min(*n*): 5, 6, or 7] between the correct and incorrect as well as ascending and descending items, respectively. Children responded by a button press of the dominant hand (index finger for “yes,” middle finger for “no”). The paradigm was programmed on E-Prime (Version 2, Psychology Software Tolls Inc., USA) and answers were recorded by an MRI compatible response box (Lumina Respond Pad, Cedrus Corporation, USA). Reaction times (RT) smaller than 300 ms and misses were not included in the analyses of the paradigm.

### Image acquisition

MRI data were acquired on a 3T General Electric Signa Scanner (GE Medical Systems, USA) using an 8-channel head coil. Whole brain functional images were acquired interleaved with a gradient echo EPI sequence [36 slices, slice thickness (ST) = 3.4 mm, no interslice skip, matrix size (MS) = 64 × 64, field of view (FOV) = 220 × 220 mm, in-plane resolution = 3.4 × 3.4 mm, flip angle (FA) = 45°, echo time (TE) = 31 ms, repetition time (TR) = 2100 ms]. Additionally, a T1-weighted structural image was obtained with a fast spoiled gradient echo sequence (3D FSPGR, ST = 1 mm, no interslice skip, MS = 256 × 192, FOV = 240 × 192 mm, FA = 20°, TE = 2912 ms, TR = 9972 ms).

Participants were carefully instructed and supplied with hearing protection before entering the scanner. To minimize head motion, the head was stabilized with padding.

### Data analysis

#### Behavioral data

Behavioral data was statistically analyzed with SPSS (Version 20). To assess group differences parametric *t*-tests for independent samples or, if data deviated from normal distribution, non-parametric Kolmogorov-Smirnov-Z-test were performed. In the cases were the assumption of homogeneity of variance was violated, we adjusted the degrees of freedom using the Welch-Satterthwaite method. A mixed-model ANOVA with time (baseline/follow-up) as within-subject factor and group (DD/TD) as between-subject factor was conducted to examine developmental effects. Effect sizes are reported as Cohen's d for *t*-tests and partial η^2^ for the mixed-model ANOVA. As suggested by Cohen (1988) effect sizes are interpreted as small (*d* = 0.2, η^2^ = 0.01), medium (*d* = 0.5, η^2^ = 0.06) or large (*d* = 0.8, η^2^ = 0.14).

#### fMRI data

##### fMRI motion

For each subject the motion finger print according to Wilke (2012) was calculated. Total displacement, a vector combining the measures of translation (x, y, and z) and rotation (pitch, roll, and yaw), was used to check if there is a difference in motion between the baseline and the follow-up measurement and TD and DD group, respectively. In more detail, the motion fingerprint provides a total displacement value and a scan-to-scan displacement value for each volume. For each subject the mean total displacement (td) and mean scan-to-scan displacement (sts) over the time series were calculated (values are reported in mm).

Groups did not differ at the baseline in td (DD: 0.34-2.10, Median = 0.66, TD: 0.43–2.34, Median = 0.98, Kolmogorov-Smirnov-Z = 1.02, *p* = 0.17, two-sided) or sts (DD: 0.06–0.41, Median = 0.14, TD: 0.07–0.73, Median = 0.13, Kolmogorov-Smirnov-Z = 0.48, *p* = 0.83, two-sided). Also at follow-up, we did not find any differences between the groups for td (DD: 0.44–1.13, Median = 0.70, TD: 0.31–1.28, Median = 0.90, Kolmogorov-Smirnov-Z = 0.81, *p* = 0.40, two-sided) and sts (DD: 0.05–0.19, Median = 0.06, TD: 0.05–0.10, Median = 0.06, Kolmogorov-Smirnov-Z = 0.26, *p* = 0.95, two-sided).

Between the baseline (0.34–2.34, Median = 0.91) and follow-up (0.31–1.28, Median = 0.75) no difference was found for td (Wilcoxon Signed Ranks Test, *z* = −1.39, *p* = 0.17, two-sided). However, the sts displacement was significantly higher for the baseline (0.06–0.73, Median = 0.13) compared to the follow-up measurement (0.05–0.19, Median = 0.06, Wilcoxon Signed Ranks Test, *z* = −3.53, *p* < 0.001, two-sided). Therefore, it is unlikely that motion affects the results of the group comparison, but it might impact the statistical power of the developmental comparison of the present study.

##### fMRI preprocessing

The data were analyzed by means of Statistical Parametric Mapping (SPM8, Wellcome Trust Centre for NeuroImaging, UK) running under Matlab (Release 2012b, The MathWorks Inc., USA).

Three dummy scans, acquired to stabilize magnetization at the beginning of the scan, were excluded from the analysis. Then the subjects' functional scans were realigned with rigid body transformations using the mean image as a reference scan. Six motion parameters (translation in x, y, and z direction as well as rotation in pitch, roll and yaw) were stored and included later in the analysis to control for motion. The mean functional image was then coregistered to the subjects' T1-weighted anatomical scan. In a next step, the individual anatomical scan was segmented into gray and white matter according to tissue probability maps of a pediatric atlas (NIH Paediatric Database) (Fonov et al., 2009, 2011). The parameters from the coregistration and segmentation were applied to the functional scans to normalize images into MNI (Montreal Neurological Institute) space. Finally, the functional images were smoothed with a Gaussian kernel of 6 mm FWHM (full width half maximum).

##### fMRI statistics

The first level analysis was performed using a mass-univariate approach based on the GLM. The time series from each subject were modeled with an event related design for the experimental and control condition using a canonical HRF (hemodynamic response function). The subjects' motion parameters were entered as additional regressors. Slow signal drifts and serial correlations were accounted for by using a high-pass filter of 180 s and a first level autoregressive model during maximum-likelihood estimation of the GLM parameters.

At the group level, a full factorial analysis with the factors group and time as well as IQ as a covariate was conducted for the contrast experimental-control condition. For the factor time (repeated measurement), within-subjects correlations were accounted for by estimating the covariance and accordingly adjusting the statistics and degrees of freedom during inference.

Statistical results are shown at *p* < 0.001, corrected for multiple comparisons using a cluster-extent threshold of *k* ≥ 19 voxels (513 mm^3^) or at *p* < 0.005 and *k* ≥ 22 (594 mm^3^). According to Slotnick (2008), the spatial autocorrelation of the data was estimated. Then a Monte Carlo simulation was run with 10'000 iterations, using a type I error voxel activation probability of 0.001, and an estimated FWHM as a Gaussian smoothing kernel in order to derive the cluster extent threshold yielding the desired correction for multiple comparisons at a *p* < 0.05 level (Slotnick, 2004).

Anatomical localization of the fMRI results was attained through the SPM Anatomy Toolbox (Eickhoff et al., 2005, 2007) and is reported in MNI coordinates.

## Results

### Behavioral data

Groups did not differ in terms of age, gender and handedness (Table 1).

#### Diagnosis of DD and general numerical abilities

Numerical abilities differed significantly between DD and TD children at baseline [ZAREKI-R *t*_(10.86)_ = −12.12, *p* < 0.001, *d* = −4.08] and follow-up [BASIS-MATH 4–8 *t*_(26)_ = −7.80, *p* < 0.001, *d* = −3.06; Table 1]. Every child in the DD group scored under the threshold value of 67 points in the BASIS-MATH and therefore still fulfilled the diagnostic criteria for DD at the follow-up. Moreover, the BASIS-MATH data revealed that the DD group differed significantly in all difficulty levels of the test (all *p* < 0.001), showing a substantial deficit in the very basic arithmetical skills at a mean age of 14 years.

Not surprisingly, at both time points DD children also performed significantly worse than the TD group in the tests measuring numerical skills at a peer level [Arithmetic subtest of the WISC-III at baseline: *t*_(15.73)_ = −3.34, *p* = 0.004, *d* = −1.68; Quantity Comparison subtest of the KFT 4–12+R at follow-up: *t*_(22)_ = −6.90, *p* < 0.001, *d* = −2.94; Table 1].

#### Number line task

The number line task 0–100 differed slightly between baseline and follow-up assessment, which is why the following results must be interpreted carefully (see also Materials and Methods section). A mixed-design ANOVA with time as within-subject factor and group as between-subject factor showed a significant effect of group [*F*_(1, 17)_ = 13.02, *p* = 0.002, η^2^ = 0.434] for the number line 0–100 (Table 2). Children with DD placed the numbers further away from the correct position compared to the TD group. There was also a significant main effect of time [*F*_(1, 17)_ = 26.42, *p* < 0.001, η^2^ = 0.609], showing that accuracy increased with development. Finally, the significant interaction time by group indicated that DD children improved more over time than TD children [*F*_(1, 17)_ = 5.44, *p* = 0.032, η^2^ = 0.243]. The number line test 0–1000, performed at the follow-up assessment, also revealed lower performance for the DD than the TD group (Kolmogorov-Smirnov-Z *z* = 1.51, *p* = 0.011, *d* = 1.21) (Table 2). Regarding the measured RT at the follow-up assessment, no significant differences could be found in the number line tasks between groups [number line 1–100: *t*_(26)_ = −1.15, *p* > 0.05, *d* = −0.45; number line 1–1000: *t*_(26)_ = −0.97, *p* > 0.05, *d* = −0.38].

**Table 2:** Behavioral results for the spatial representation of numbers (number line), basic arithmetic operations (addition and subtraction), and the fMRI paradigm.

**Behavioral measure**		**Baseline**		**Follow-up**							
		**DD**	**TD**	**DD**	**TD**	**Effect of group**		**Effect of time**		**Interaction**	
	**N^1^**	***M (SD)***	***M (SD)***	***M (SD)***	***M (SD)***	**Test- statistic**	***p***	**Test- statistic**	***p***	**Test- statistic**	***p***
**NUMBER LINE**											
1–100 [% distance]	9/10	10.6 (4.3)	5.6 (1.2)	5.1 (1.2)	3.5 (2.2)	13.02^a^	0.002^**^	26.42^a^	< 0.001^***^	5.44^a^	0.032^*^
1–1000 [% distance]	17/11			9.4 (5.8)	4.1 (2.3)	1.51^b^	0.011^*^				
**BASIC ARITHMETIC OPERATIONS**											
Addition Accuracy	9/10	14.2 (4.3)	19.2 (0.9)			−3.43^c^	0.008^**^				
	17/11			16.0 (3.8)	18.7 (1.4)	−2.30^c^	0.030^*^				
Subtraction Accuracy	9/10	12.7 (2.9)	17.4 (1.8)			1.72^b^	0.001^**^				
	16/11			13.0 (4.0)	17.6 (2.4)	−3.79^c^	0.001^**^				
Addition RT [s]	17/11			19.4 (11.1)	13.2 (2.9)	2.20^c^	0.041^*^				
Subtraction RT [s]	16/11			18.2 (4.9)	14.4 (3.2)	2.42^c^	0.023^*^				
**FMRI PARADIGM**											
Accuracy [%]	16/10	74.9 (12.8)	84.5 (11.2)	91.9 (6.2)	95.4 (4.2)	4.30^a^	0.049^*^	40.85^a^	< 0.001^***^	1.95^a^	0.176
RT [ms]	16/10	1718 (372)	1615 (280)	1331 (278)	1261 (255)	1.91^a^	0.180	50.45^a^	< 0.001^***^	0.43^a^	0.517

1 *Number of subject per group DD/TD*.

a *Mixed-design ANOVA*.

b *Kolmogorov-Smirnov-Z Test*.

c *t-Test*.

* *p < 0.05*,

** *p < 0.01*,

*** *p < 0.001*.

#### Basic arithmetic operations

For the basic arithmetic operations, *t*-test revealed significant differences between DD and TD children. At baseline and follow-up, TD children solved more addition [baseline: *t*_(8.67)_ = −3.43, *p* = 0.008, *d* = −2.33; follow-up: *t*_(26)_ = −2.30, *p* = 0.030, *d* = 0.90] and subtraction problems correctly [baseline: Kolmogorov-Smirnov-Z *z* = 1.72, *p* = 0.001, *d* = 1.96, follow-up: *t*_(24.67)_ = −3.79, *p* = 0.001, *d* = −1.53; Table 2]. The measured RTs at follow-up show that children with DD took longer to solve the addition [*t*_(19.19)_ = 2.20, *p* = 0.041, *d* = 0.91] and the subtraction problems [*t*_(24.99)_ = 2.42, *p* = 0.023, *d* = 0.97] compared to their peers (Table 2).

#### Intelligence quotient

All participants reached normal range of intelligence during both assessments (IQ range baseline: 93–125; follow-up: 92–122). However, groups differed significantly in the estimated general IQ [baseline: WISC-III *t*_(26)_ = −4.65, *p* < 0.001, *d* = −1.82; follow-up: WISC-IV *t*_(26)_ = −4.41, *p* < 0.001, *d* = −1.73; Table 1]. Differences in IQ scores between a group of children with learning disabilities and a control group are often reported in the literature (Geary et al., 2000; Willcutt et al., 2013). One reason for this is that IQ-tests are not independent from numerical skills. The IQ was not entered as a covariate in the subsequent behavioral analysis, since IQ is not independent from the effects of interest (Miller and Chapman, 2001; Dennis et al., 2009; Field, 2009).

#### Attention, reading and working memory

To match the groups for comorbid attention deficit and hyperactivity disorder (ADD/ADHD) and dyslexia, the TAP and the SLRT-II were performed. Groups did not differ significantly in any measurement of attention or reading performance (Table 1). Regarding working memory, subjects showed at baseline and follow-up comparable results in the verbal and visuo-spatial memory component. The only significant difference was found in visuo-spatial working memory at the follow-up assessment due to lower performance of children with DD compared to TD (Kolmogorov-Smirnov-Z *z* = 1.16, *p* = 0.039, *d* = −0.76) (Table 1).

#### Behavioral results from fMRI task

A mixed-design ANOVA with time as within-subject factor and group as between-subject factor was calculated. For accuracy, the ANOVA revealed a significant effect of time [*F*_(1, 24)_ = 40.85, *p* < 0.001, η^2^ = 0.63], showing that children were better able to solve the task with increasing age (Table 2). Furthermore, the DD group performed significantly worse than the TD group [*F*_(1, 24)_ = 4.30, *p* = 0.049, η^2^ = 0.152]. This significant difference arises from their lower performance in the number order task [*F*_(1, 24)_ = 4.54, *p* = 0.044, η^2^ = 0.159], as performance in the control task was comparable between groups [*F*_(1, 24)_ = 1.66, *p* = 0.209, η^2^ = 0.065]. The group by time interaction was not significant [*F*_(1, 24)_ = 1.95, *p* = 0.176, η^2^ = 0.075]. For RT, no effects of group [*F*_(1, 24)_ = 1.91, *p* > 0.05, η^2^ = 0.074] or interaction between time and group [*F*_(1, 24)_ = 0.43, *p* > 0.05, η^2^ = 0.018] was evident. However, children solved the task faster at the second assessment point [*F*_(1, 24)_ = 50.45, *p* < 0.001, η^2^ = 0.678; Table 2].

### fMRI results

**Figure 3:**
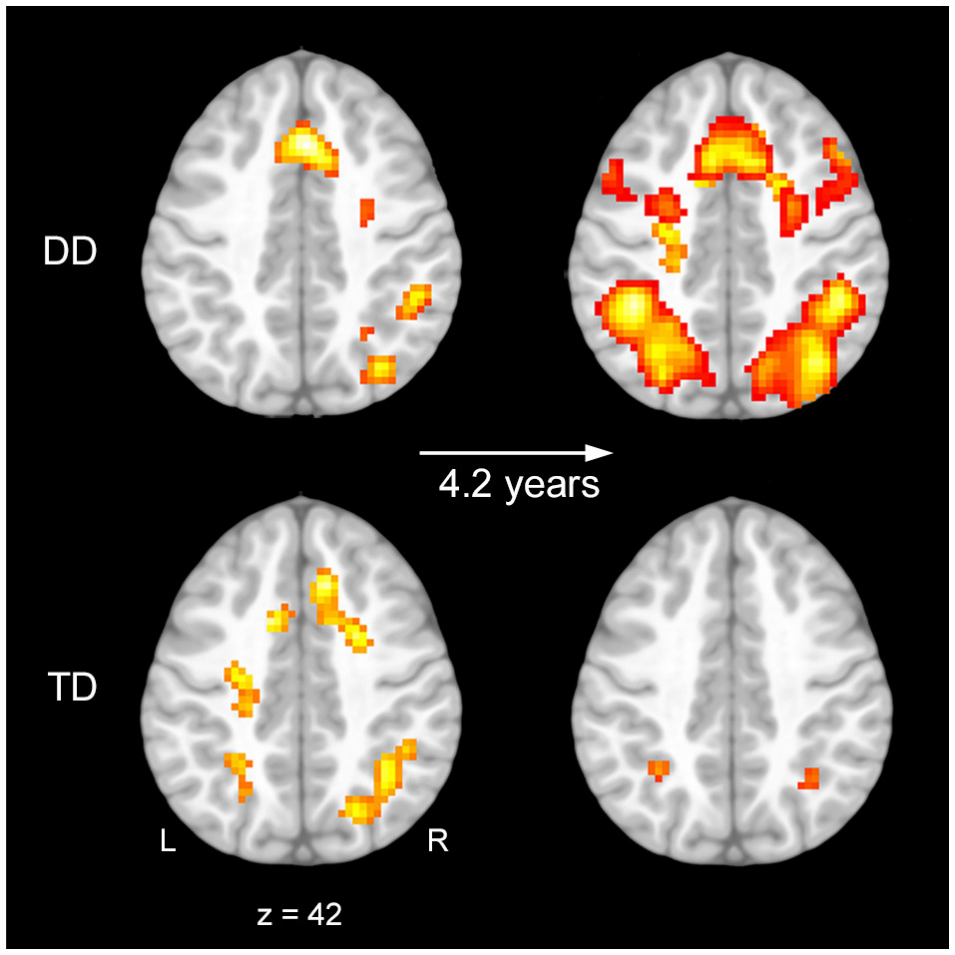
Activation at baseline and follow-up. Task related brain activation shown on a pediatric template (Fonov et al., 2009, 2011) for the contrast numerical order vs. control task at baseline **(Left)** and follow-up **(Right)** for children with developmental dyscalculia (DD) **(Upper)** and typically developing (TD) children **(Lower)** (*p* < 0.01, *k* ≥ 24, cluster-extent corrected).

Analysis of the task (experimental minus control condition) revealed bilateral parietal activation in TD children at baseline and follow-up. DD children showed at baseline only right lateralized activation in parietal regions (Figure 3, Tables 3, 4).

**Table 3:** Brain areas that showed significant activation for the numerical order vs. control task from dyscalculic and typically developing children at the baseline assessment (*p* < 0.01, *k* ≥ 24, cluster-extend corrected).

**Region**	**Cluster**	**Peak**	**Peak MNI**		
	**size**	***t*-value**	**coordinates**		
			**x**	**y**	**z**
**BASELINE ASSESSMENT**					
**Dyscalculic children**
L superior medial gyrus	203	5.21	1	22	42
Cerebellar vermis	143	4.65	1	−56	−36
L cerebellum	131	4.56	−32	−59	−36
R middle occipital gyrus	87	4.42	31	−71	39
R cerebellum	178	4.27	34	−56	−42
R intraparietal sulcus	173	4.12	43	−41	36
R precentral gyrus	43	3.64	49	1	24
R middle frontal gyrus	153	3.63	31	−2	54
Cerebellar vermis	34	3.48	4	−47	−15
R insula	35	3.33	31	19	6
R calcarine gyrus	71	3.26	31	−62	6
**Typically developing children**					
L precentral gyrus	175	4.46	−29	−14	48
L middle occipital gyrus, intraparietal gyrus	182	4.21	−26	−71	24
R superior occipital gyrus, intraparietal gyrus	276	4.20	22	−74	36
Middle cingulate cortex, SMA	630	4.17	−14	10	39
L insula	55	3.88	−35	13	6
R inferior frontal gyrus	25	3.64	52	7	18
L calcarine gyrus	83	3.55	−17	−80	9
Thalamus	27	3.04	−2	−20	6

**Table 4:** Brain areas that showed significant activation for the numerical order vs. control task from dyscalculic and typically developing children at the follow-up assessment (*p* < 0.01, *k* ≥ 24, cluster-extend corrected).

**Region**	**Cluster**	**Peak**	**Peak MNI**		
	**size**	***t*-value**	**coordinates**		
			**x**	**y**	**z**
**FOLLOW-UP ASSESSMENT**					
**Dyscalculic children**
L cerebellum	7531	6.98	−32	−62	−36
L inferior parietal lobe		5.44	−41	−44	45
R middle occipital gyrus, supramarginal gyrus	1190	6.20	34	−68	39
R inferior temporal gyrus	61	3.95	58	−44	−15
R calcarine gyrus	199	3.91	31	−77	3
L inferior frontal gyrus	35	3.44	−47	40	−6
**Typically developing children**					
L caudate nucleus	179	5.31	−17	−14	27
L hippocampus	155	4.83	−29	−65	0
L cerebellum, cerebellar vermis	252	4.59	1	−41	−42
L thalamus	103	4.22	1	−20	6
N/A	52	4.12	1	7	18
L cerebellum	63	3.95	−26	−65	−33
R hippocampus	40	3.88	25	−38	15
R caudate nucleus	75	3.70	10	−14	21
L intraparietal sulcus	25	3.20	−32	−53	42
R intraparietal sulcus	25	2.98	34	−59	45

#### fMRI group differences

At baseline, no significant difference between groups was found at the statistical threshold of *p* < 0.001.

**Figure 4:**
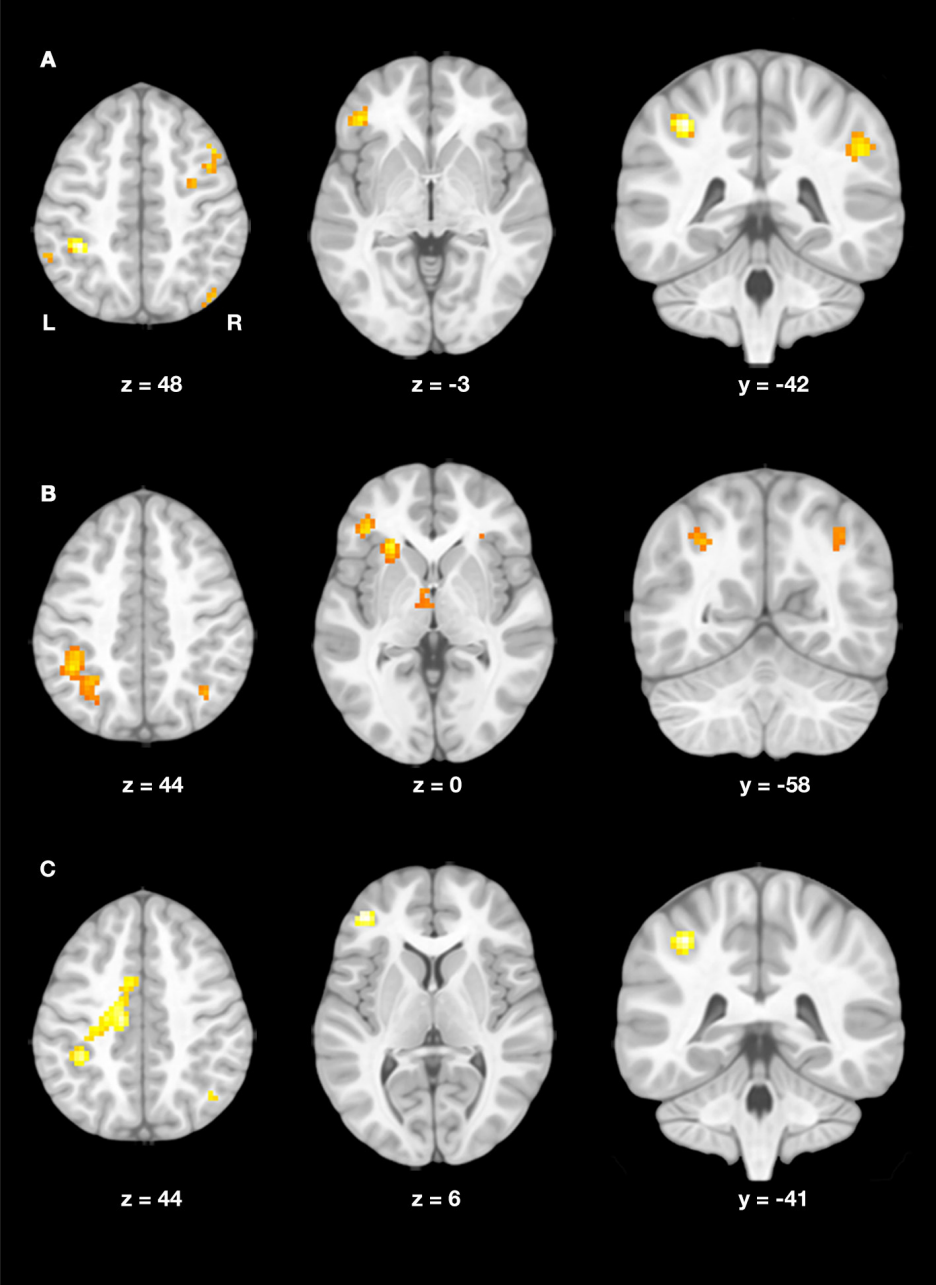
**(A)** Group differences at follow-up. Increased activation in the dyscalculic compared to the typically developing group at the follow-up assessment (*post-hoc t*-test for the contrast DD vs. TD, *p* < 0.001, *k* ≥ 19, cluster-extent corrected). **(B)** Developmental increase in DD. Increase in brain activation in the dyscalculic group over developmental time (*post-hoc t*-test for the contrast follow-up vs. baseline, *p* < 0.005, *k* ≥ 22, cluster-extent corrected). **(C)** Negative interaction. Activation increase over time was more pronounced in children with dyscalculia compared to typically developing children (group by time interaction, *p* < 0.005, *k* ≥ 22, cluster-extent corrected).

At follow-up, two-sample *t*-tests revealed significant differences between children with DD and controls (Figure 4A, Table 5). Children with DD showed increased activation in frontal areas including bilateral middle frontal gyri (MFG) and the left inferior frontal gyrus (IFG). In the parietal lobe, more activation in the bilateral angular gyri (AG), extending into the supramarginal gyri (SMG) and the left IPS was found. TD children did not show any increased activation compared to children with DD at follow-up (*p* < 0.001).

**Table 5:** Brain areas that showed significant activation for the contrast dyscalculic vs. typically developing children at the follow-up assessment (*p* < 0.001, *k* ≥ 19, cluster-extend corrected).

**Region**	**Cluster**	**Peak**	**Peak MNI**		
	**size**	***t*-value**	**coordinates**		
			**x**	**y**	**z**
**GROUP DIFFERENCES AT THE FOLLOW-UP ASSESSMENT**					
L intraparietal sulcus	37	5.56	−38	−41	48
R middle frontal gyrus, precentral gyrus	65	4.39	43	19	45
L middle frontal gyrus	36	4.38	−29	4	63
R angular gyrus	55	4.33	40	−71	42
L inferior frontal gyrus	49	4.15	−44	37	−3
R supramarginal gyrus	45	4.14	49	−44	33
L angular gyrus, supramarginal gyrus	49	3.82	−53	−53	42
L angular gyrus	21	3.40	−38	−68	42

#### fMRI developmental effects

Developmental changes took place in the DD group, showing increased activation in the basal forebrain and the left insula at *p* < 0.001. At a threshold of *p* < 0.005, additional activation increases in the bilateral IPS, right insula, left IFG, left parahippocampal gyrus (PHG) and left thalamus were observed (Figure 4B, Table 6). No decrease in activation was found in the DD group over development.

**Table 6:** Brain areas that showed significant developmental changes in children with developmental dyscalculia and the negative interaction group by time (*p* < 0.005, *k* ≥ 22, cluster-extend corrected).

**Region**	**Cluster**	**Peak**	**Peak MNI**		
	**size**	***t*-value**	**coordinates**		
			**x**	**y**	**z**
**DEVELOPMENTAL INCREASE IN DD**					
Basal forebrain	137	4.96	−2	−11	−18
L insula	78	4.35	−29	25	−6
L intraparietal sulcus	176	3.81	−23	−50	33
R putamen, insula	61	3.75	28	7	−12
L parahippocampal gyrus	34	3.62	−26	−14	−27
L inferior frontal gyrus	39	3.59	−41	34	−3
L thalamus	32	3.31	1	−17	6
R intraparietal sulcus	23	3.17	37	−62	45
**NEGATIVE INTERACTION GROUP × TIME**					
L inferior frontal gyrus	60	3.99	−41	43	6
L inferior parietal sulcus	58	3.96	−38	−41	48
L middle cingulum	134	3.90	−38	−29	33
L hippocampus	24	3.68	−29	−14	−30
R middle occipital gyrus, angular gyrus	22	3.16	34	−62	36

TD children did not show any increase or decrease in activation over development at the statistical threshold of *p* < 0.005.

The negative interaction time by group indicated that the activation increase over time was more pronounced in children with DD than in TD children. The left IFG was the only region showing interaction effects at the higher cluster-extent threshold (*p* < 0.001). A lower threshold (*p* < 0.005) revealed activation in similar regions to the *t*-test in DD over development, namely in the left middle cingulum extending into somatosensory area, left IPS, left hippocampus, and right AG (Figure 4C, Table 6).

In order to investigate the developmental effects further, a regression analysis was performed with 14 fMRI data sets, comprised of both baseline and follow-up scans. Each subjects' activation increase over time, for the contrast experimental minus control condition (interaction time point x condition), and the number of the correctly solved basic arithmetic operations (addition and subtraction) at baseline were included in the analysis. Results revealed a negative correlation between the activation increase over time and the number of correctly solved subtractions and additions at the baseline assessment (cluster-extent corrected *p* < 0.001). This indicates that children who solved fewer arithmetic problems correctly at baseline showed more activation increase over time in bilateral cingulate cortex extending into right frontal gyri and left supplementary motor area (SMA), bilateral insular lobe extending bilaterally into putamen and caudate nucleus, left inferior (IFG) and middle frontal gyrus (MFG), left superior temporal gyrus (STG) and right cerebellum. In the parietal lobe, broad activation increases were found in bilateral angular gyri (AG) extending into inferior parietal lobe and intraparietal sulcus (IPS), and bilateral precuneus (see Figure S1).

## Discussion

In the present longitudinal study, we investigated the neuro-functional development of children with and without DD by means of neuropsychological tests and fMRI. In line with previous studies, we found that children with DD improved over time, but nonetheless showed persistent deficits in number processing and arithmetical skills when compared to their peers.

Brain imaging results revealed an increase in frontal and parietal brain activation over time in children with DD. In contrast, results of TD children point to a stable activation pattern over development. Furthermore, a lower performance in basic arithmetic operations correlates with a more pronounced increase in the fronto-parietal network over time.

### Deficient numerical processing and aberrant neural networks

As hypothesized, we found considerable deficits in number processing and arithmetic abilities in children with DD compared to a peer group. The more pronounced inaccuracy in a number line task is typically found in dyscalculics and is consistent with a large body of research findings (Geary et al., 2008, 2012; Landerl, 2013). In addition, the accuracy in a number line task is thought to reflect a better representation of quantity (Siegler and Booth, 2004; Ebersbach et al., 2008). Therefore, our data point to a deficient mental number line representation in 9 and 14-year old dyscalculic children. Consistent with the study from Piazza et al. (2010), the children with DD performed at the same level as the control group when 4-years younger. Given that numerical magnitude representation further influences arithmetical learning, it is in good agreement with earlier studies (Booth and Siegler, 2008) that our DD group also showed poor performance in basic addition and subtraction problems.

Regarding brain activation, group differences were evident at the follow-up assessment. Children with DD showed increased activation in frontal (MFG, IFG) and parietal (AG, left IPS) regions of the numerical network compared to their peers. This is in contradiction with studies reporting reduced activation in the parietal key regions for numeracy. However, our findings are in line with several studies, who found increased activation in fronto-parietal regions of DD children (Kaufmann et al., 2009b; Kucian et al., 2011b; Iuculano et al., 2015; Rosenberg-Lee et al., 2015). Similar results were further reported in the meta-analysis by Kaufmann et al. (2011). The authors suggested, that the increased IPS and postcentral activation reflects the recruitment of finger-based number representation in DD children, which might also be the case in our study.

At baseline, we did not find any differences in the activation pattern of the groups. A reason for this is that we chose a rather strict significance level to report our results. When lowering the statistical threshold activation differences in occipito-parietal, temporal and frontal regions could be detected, which are in line with those reported in the literature (e.g., Kaufmann et al., 2009b; Kucian et al., 2011b).

### Typical and atypical development

Consistent with our expectations, TD children showed a growing proficiency in number processing with development, as seen in a significant improvement in the number line task. DD children also did not stagnate in their development, exhibiting a decrease in error rates when placing numbers on a number line. In fact, our results showed that the dyscalculic's number line performance improved more over time than that of the TD children. This result is consistent with other findings of long-term studies (Geary et al., 2012; Landerl, 2013). However, even when the gap between the typical and atypical development in the mental number line decreases, children with DD always performed significantly lower than their peers. Our results confirm findings from earlier studies (Shalev et al., 1998, 2005) and the result from a systematic review (Nelson and Powell, 2017) showing that number representation in DD is deficient and delayed in development. In addition, children with DD still showed substantial deficits in simple arithmetic through the entire study. This result supports the importance of effective and efficient ordinal and magnitude number processing abilities in the development of arithmetical skills (Booth and Siegler, 2008).

The brain activation patterns of TD children revealed no significant difference over the examined time. This result seems surprising, considering findings from earlier studies, who showed an age related activation increase in the IPS and a decrease in frontal areas during magnitude processing (Ansari et al., 2005; Ansari and Dhital, 2006). However, it is important to note that most of the studies compare numeracy-established adults with developing children and therefore assume linearity in development. It might be that some of the mentioned changes occur only at specific periods in development. Moreover, the brain activation pattern from our results is in line with results from a study using the same task (Kucian et al., 2011a). Our findings are further consistent with Kucian et al. (2008), revealing no differences comparing children over a 3-year period, but finding changes between children and adults. Along with the increasing proficiency on the behavioral level, our results speak for a consistent and well working number processing network in TD children. Importantly, the present results do not exclude the possibility that the number processing network continuously develops and refines in typical development over time. Furthermore, these results must be interpreted cautiously and confirmed with bigger group sizes.

Interestingly, children with DD showed a remarkable activation increase in the entire fronto-parietal network over the observed period of development. The growth of activation in the basal forebrain, bilateral insula and bilateral IFG is in good agreement with the literature, indicating that these regions play a crucial role in working memory, attention, and cognitive control. Together with the better performance in the fMRI task, this finding supports the notion that children with DD constantly use domain-general regions to a larger extent, reflecting the higher cognitive demands induced by the task. Besides, children with DD showed an activation increase in the bilateral IPS over developmental time. It is further worth pointing out, that in children with DD the activation increase in the left IPS was much greater than in the right IPS, whilst TD peers showed stable bilateral IPS activation over time. Findings show that activation changes with growing proficiency in (symbolic) number representation in the left IPS and is stable over development in the right IPS (Vogel et al., 2015). In context with the improvement in the fMRI paradigm and the catch-up in the number line task, our results lend support to a stronger use of number-specific areas in children with DD. This is in line with the results of the regression analysis, indicating that children who solved fewer arithmetic problems correctly at baseline showed more activation increase over time. Furthermore, the negative interaction also revealed activation in parietal number-specific regions and frontal domain-general regions, indicating that the developmental changes were more pronounced in children with DD. This mirrors the results from our behavioral data and previous studies, showing that the gap between TD and DD performance diminishes over development (Geary et al., 2012; Landerl, 2013).

To our knowledge, no neuro-imaging long-term studies exist in the field of dyscalculia, but results from studies with dyslexic children also showed differences in the development of the neural reading system. Comparable to our findings, age related increases are seen in domain-specific occipito-temporal regions but also in domain-general regions (left IFG) (Shaywitz et al., 2007). Furthermore, Rosenberg-Lee et al. (2011) looked at brain maturation processes between 2nd and 3rd grades during arithmetic problem solving. In line with our results, better behavioral performance and a significant increase in activity were observed in the right superior parietal lobe, IPS and AG, PHG, and frontal regions from grade 2 to 3. Based on these activation increases, which have been associated with initial stages of learning, the developmental effects in our DD group might also reflect neural maturation processes.

To summarize, our results support the notion that TD children have a well-functioning number processing network, and therefore showed only subtle developmental effects over the examined time. Dyscalculic children, however, showed age-related changes in frontal areas of the brain. These can be related to compensatory mechanisms or different but less effective task solving strategies, which are often observed in children with DD. Secondly, the increase in domain-specific parietal areas, hints to maturation or delayed development of number processing areas. Although these findings are promising, it is important to note that children with DD did not fully catch up to their peer group in numerical-arithmetical skills and showed less focused activation patterns, underscoring that the deficiencies do not fully vanish with time.

### Methodological considerations

To our knowledge this is the first longitudinal study looking at neural development in children with and without DD. The lack of other longitudinal studies in DD might arise from several reasons. Firstly, longitudinal fMRI studies in children are especially prone to high drop-out due to more movement artifacts and braces. This was also the case in our study and the reason why we have unequal and small sample sizes. For this reason, our results (in particular the results from the TD children) should be interpreted with caution. However, the same main results were obtained when evaluating the study with equal group sizes revealing that our results are stable and not based on differences in group size (see Supplementary Material and Figures S2–S6). Furthermore, in order to check the statistical power of our findings, we conducted *post-hoc* power analyses (G^*^Power; Faul et al., 2007) for the significant main results of the behavioral data with α = 0.05, and the effect and sample sizes as reported for the specific statistical test (see Results section, Tables 1, 2). For most of the tests we reached good statistical power (1−β ≥ 0.80). However, for the interaction of the number line test 1–100 and the effect of group in the accuracy of the fMRI paradigm we detected a power of 0.60 and 0.51, respectively. Thus, the likelihood that these results reflect true effects is reduced. In addition, power analyses for the main effects of the fMRI data were conducted by means of the software package fMRIpower (Mumford and Nichols, 2008). This method estimates power for detecting significant activation within specific regions of interest, with the assumption that the planned studies will have the same number of runs per subject, runs of the same length, similar scanner noise characteristics, and data analysis with a comparable model (Mumford and Nichols, 2008). For this purpose, post-training data from Kucian et al. (2011a) (which were acquired with the same fMRI paradigm on the same MR-Scanner, but were not included in the present longitudinal study, see also Materials and Methods section) were used as “pilot data” for the power analysis. The power analyses were carried out for each of the regions of the automated anatomical labeling (aai) roi mask with α = 0.05 and the sample sizes as reported for the specific contrast of the present study. For the *group differences at the follow-up assessment* (Figure 4A, Table 5), power estimates between 11 and 62% were obtained for the brain areas that showed significant activation, with the highest power estimate of 62% observed in the left IPS and angular gyrus. Similarly, the brain areas that showed significant *developmental changes in children with DD* (Figure 4B, Table 6), reached power estimates between 18 and 79%. The highest values of 70 and 79% of power were detected for the left and the right IPS, respectively, whilst lower power estimates were reached for the frontal areas of the brain. Despite the fact that more subjects would be necessary to increase the power of the present study, the results of the conducted power analyses reveal that the power estimates for the numerical key areas are already near to the desired power of 80% and therefore likely show true effects.

Secondly, the choice of the fMRI paradigm, especially in longitudinal studies, is constrained by the requirements that it must be feasible for children with DD (performance over chance level) and not too easy for TD children (ceiling effects). An adaptation of the difficulty level of the task results in a loss of comparability over time, which we wanted to avoid. As a consequence, ceiling effects might have led to a loss of behavioral group differences at the follow-up.

Thirdly, longitudinal study designs are very time consuming regarding (re-)recruitment and maintenance of the participant's motivation. Thus, developmental questions are in many cases examined by more time-efficient methods such as cross-sectional designs. Importantly, cross-sectional designs do not take into account inter-individual differences to the same extent as longitudinal designs. Furthermore, most cross sectional-studies compare adults and children and might therefore miss an opportunity to capture the full developmental trajectory. We think that our results are promising and provide an important contribution to the understanding of the typical and atypical development of number processing, but further work is needed to verify our findings and strengthen the understanding of developmental trajectories.

Despite these methodological considerations, our findings suggest a continuation in the neural development of number representation in children with DD, whereas the neural network for simple ordinal number estimation seems to be stable or show only subtle changes over time in TD children. Furthermore, our results shed light on the behavioral and neural trajectories in dyscalculia and emphasize the importance of longitudinal studies for the understanding of development. This knowledge contributes to the understanding of numeracy and might therefore be meaningful for education and implementation of therapy and support of children with difficulties in mathematics.

## Author contributions

All authors have contributed and have approved the final manuscript. UMc: contributed to the design of the study, the acquisition, analysis, and interpretation of the data, and writing the manuscript; MvA: contributed to the design of the study, data interpretation and revised the manuscript; UM, EM, and RO: contributed to data interpretation and revised the manuscript; KK: contributed to the design of the study, the acquisition and interpretation of the data, and editing and revising the manuscript.

### Conflict of interest statement

The authors declare that the research was conducted in the absence of any commercial or financial relationships that could be construed as a potential conflict of interest.

## Abbreviations

ADD/ADHD: Attention Deficit and Hyperactivity Disorder
DD: Developmental Dyscalculia
fMRI: functional Magnetic Resonance Imaging
IQ: Intelligence Quotient
MNI: Montreal Neurological Institute
RT: Reaction Time
TD: Typically Developing.
