# Supplementary material for: Longitudinal Brain Development of Numerical Skills in Typically Developing Children and Children with Developmental Dyscalculia
Source: Front Hum Neurosci. 2018 Jan 4;11:629. doi: 10.3389/fnhum.2017.00629 (PMC5758587; doi:10.3389/fnhum.2017.00629)
Supplement: Supplementary file 1 [file DataSheet1.pdf]

Supplementary Material

Longitudinal brain development of numerical skills in typically developing children and children with developmental dyscalculia

Ursina McCaskey*, Michael von Aster, Urs Maurer, Ernst Martin, Ruth O'Gorman Tuura, Karin Kucian

*** Correspondence:** Ursina McCaskey: ursina.mccaskey@kispi.uzh.ch

1 Neuropsychological Test Battery for Number Processing and Calculation in Children – Revised (ZAREKI-R)

The ZAREKI-R test battery is a norm-based test conceptualized to identify children with numerical-arithmetical deficits from grades 1 to 4. The ZAREKI-R consists of 12 subtests assessing basic numerical skills as well as calculation. Each correct answer is rewarded with 1 to 2 points resulting in a total raw test score of 122 points. The test has no time limit. Criteria for DD are met if the scores in three subtests or the total test score is below the 10th percentile rank.

The 12 subtests are:

- *Enumeration:* Children have to enumerate different sets of dots (e.g. 13 dots arranged in a line, 18 dots randomly arranged). Maximal score is 5 points (1 point for each correct answer).
- *Counting backward:* Children are asked to count from a given number backwards (e.g. 22-1, 67-54). Maximal score is 4 points (2 point for perfect performance, 1 point when one mistake, no points when more than one mistake).
- *Writing numbers:* Children have to write Arabic numbers from dictation (e.g. 14, 4658). Maximal score is 8 points (1 point for each correct answer).
- *Mental calculation:* Children have to solve orally presented addition, subtraction and multiplication problems (e.g. 5+8, 32-17). Maximal scores are 8, 8 and 6 points, respectively (1 point for each correct answer).
- *Reading numbers:* The child is asked to read Arabic numbers aloud (e.g. 305, 969). Maximal score is 8 points (1 point for each correct answer).
- *Number line estimation:* Children have to position given numbers on a number line between 1 and 100 (e.g. 86). In the first part of this task, children have to choose between four possible positions. In the second part, children have to position the numbers themselves. Maximal score is 6 and 12 points, respectively (first part: 1 point for each correct answer, second part: depending on the distance to the correct position the answer is worth 1 or 2 points).
- *Digit span:* This subtest assesses the digit span forward and backward (e.g. 2-7-3-9). Maximal score is 24 points (1 point for each correct answer).
- *Magnitude comparison of spoken words*: Pairs of numbers are presented orally and the child has to judge which one is larger (e.g. eight hundred – hundred and eight). Maximal score is 8 points (1 point for each correct answer).
- *Perceptual quantity estimation:* Children have to estimate the numerosity of visually presented sets of objects (e.g. 57 balls, 89 cups). Maximal score is 5 points (1 point for each correct answer).

- *Contextual magnitude estimation:* Children have to estimate in relation to the context if a quantity represents few, average or a lot (e.g. fifteen words in a reading book, four fridges in a kitchen). Maximal score is 6 points (1 point for each correct answer).
- *Problem solving:* The child is asked to solve different story problems (e.g. Peter has 16 marbles. He has 4 marbles more than Anne. How many marbles does Anne have?). Maximal score is 6 points (1 point for each correct answer).
- *Magnitude comparison of Arabic numbers:* Pairs of numbers are presented visually and the child has to judge which one is larger (e.g. 654 or 546). Maximal score is 8 points (1 point for each correct answer).

The normative sample consists of 764 Swiss and German children from grades 1 to 4. Population mean (μ) and standard deviation (σ) of the norm as well as the mean (M) and standard deviation (SD) of the sample are reported for each grade separately.

ZAREKI-R raw score	DD M (SD)	TD M (SD)	Norm population μ (σ)
Grade 2	47 (10.8)	100 (6.9)	88 (16.2)
Grade 3	88 (7.3)	109 (5.0)	102 (7.3)
Grade 4	97 (5.4)	112 (5.0)	109 (5.7)

The mean (M), standard deviation (SD) and range of the standard scores of the ZAREKI-R are reported for the DD and TD sample.

ZAREKI-R standard scores	DD M (SD) [range]	TD M (SD) [range]	Norm population μ (σ) [range]
	5.9 (4.9) [1-18]	77 (19.1) [46-100]	50 (34.1) [1-100]

The reliability for the ZAREKI-R is high (Cronbach's alpha = .93-.97).

2 Basic Diagnosis in Mathematics Education for Grades 4-8 (BASIS-MATH 4-8)

The BASIS-MATH test battery is a criterion-referenced test conceptualized to identify children with deficits in the basic mathematical concepts from grades 4 to 8. In accordance to the WHO definition of developmental dyscalculia, the mathematical performance is measured by the criterion “not reaching mastery of the basic mathematical concepts”. The BASIS-MATH test battery is composed of three difficulty levels measuring several arithmetical abilities. The subjects have to solve 48 tasks, which base on the arithmetic and mathematical knowledge acquired in grade 1 through 4. Each correct answer is rewarded with 1 to 2 points. The test has no time limit. Criteria for DD are met if the performance is under a threshold value of 67 points (maximum score 83 points).

The difficulty levels are set as follows: *Difficulty level I:* Skills in basic arithmetic operations in the number range 0-100. These skills correspondsto the requirements of the second grade. *Difficulty level II:* Skills in basic arithmetic operations in the number range 0-1000. *Difficulty level III:* Skills in the place-value-system and understanding of the basic mathematical concepts.

The arithmetical abilities included in the test are:

- *Mental and written calculation:* Children have to solve additions, subtractions, multiplications and divisions of different difficulties (e.g. 47+36, 1000:8).
- *Subsets:* Children have to complete subsets and build part-whole-relationships (e.g. 1000 - ? = 670).
- *Counting:* The child is asked to count forward and backward in steps of two, ten and hundred (e.g. count backward in steps of ten starting at 137).
- *Decimal system:* Children have to create subsets, solve tasks regarding the place-value-system and make number line estimations (e.g. write down the number: 7 thousands, 3 tens and 15 units).
- *Problem solving:* Three story problems have to be solved (e.g. three pieces of cheesecake cost CHF 7.20. How much does one piece cost?).

The BASIS-MATH is scaled according to the one-dimensional Rasch model. The normative sample consists of 692 Swiss and German children from grades 4 to 8. The test has a high sensitivity (92%) and reliability (internal consistency Cronbach’s alpha = .92).

3 Regression Analysis

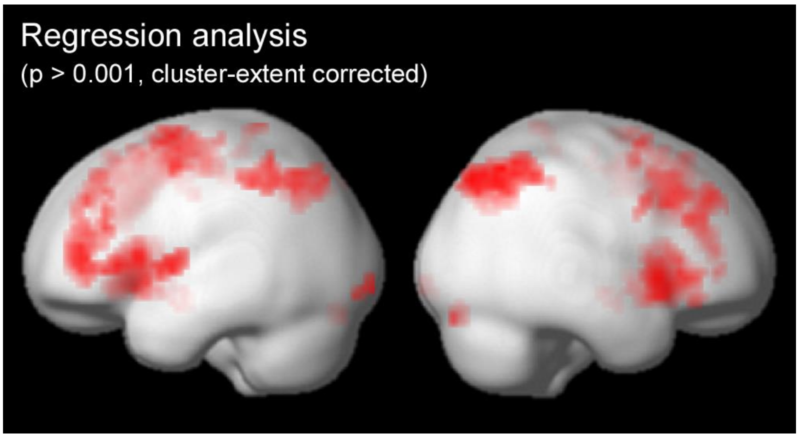

Figure S1. Regression analysis. Negative correlation between the activation increase over time and the number of correctly solved subtractions and additions at the baseline assessment in the bilateral cingulate cortex extending into right frontal gyri and left supplementary motor area (SMA), bilateral insular lobe extending bilaterally into putamen and caudate nucleus, left inferior (IFG) and middle frontal gyrus (MFG), left superior temporal gyrus (STG), right cerebellum, bilateral angular gyri (AG) extending into inferior parietal lobe and intraparietal sulcus (IPS), and bilateral precuneus.

4 Analysis with equal group sizes

A full factorial analysis with the factors group and time as well as IQ as a covariate was conducted for the contrast experimental-control condition. Only subjects with data available at baseline and follow-up were included (resulting in 11 DD and 8 TD fMRI data sets). In a second step, 3 random data sets of the DD group were excluded to obtain equal group sizes (8 fMRI data sets per group and time point).

4.1 fMRI group differences

At baseline, children with DD showed no increased activation compared to TD children at the statistical threshold of $p < .001$ (Figure S2, left side). However, TD children showed increased activation in the left insula (x = -35, y = -8, z = 6) and the left postcentral gyrus (x = -17, y = -32, z = 72) (Figure S2, right side).

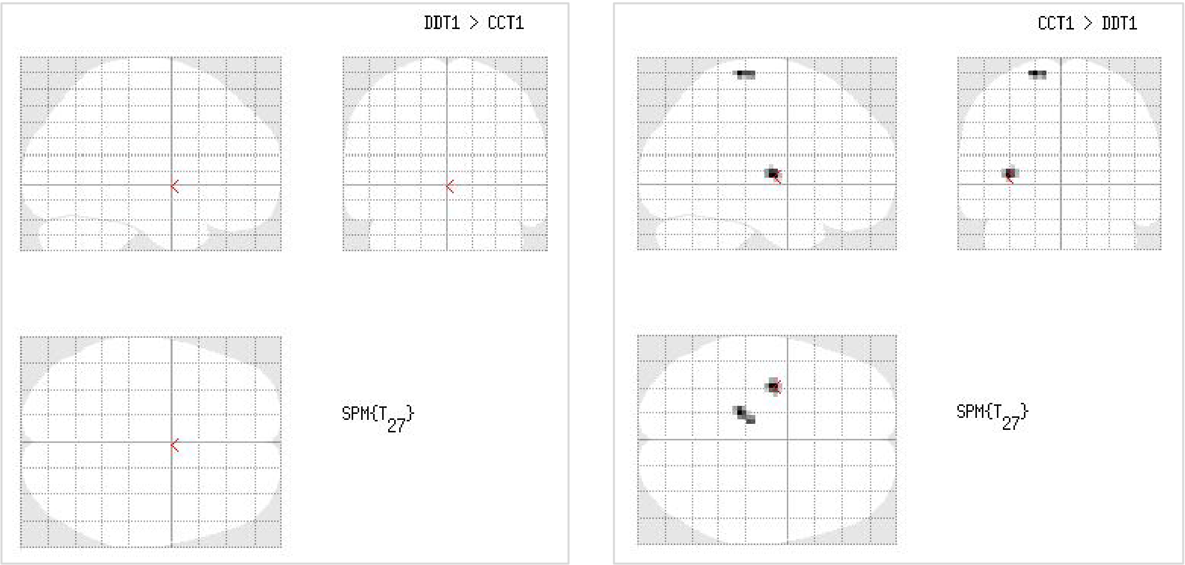

Figure S2. fMRI group differences at baseline. Left side. Glass brain showing that the contrast DD vs. TD children elicited no activation at baseline. Right side. Activation for the contrast TD vs. DD children at baseline ($p < .001$, $k \geq 19$, cluster-extent corrected).

At follow-up, two-sample t-tests revealed significant differences between children with DD and controls in similar regions as in the main analysis of the paper (right angular gyrus (AG) x = 40, y = -71, z = 42, left inferior parietal cortex (IPC) x = -56, y = -53, z = 45 and left intraparietal sulcus (IPS) x = -38, y = -41, z = 48) (Figure S3). TD children showed increased activation in the vermis of the cerebellum (x = -2, y = -41, z = -39) (Figure S4).

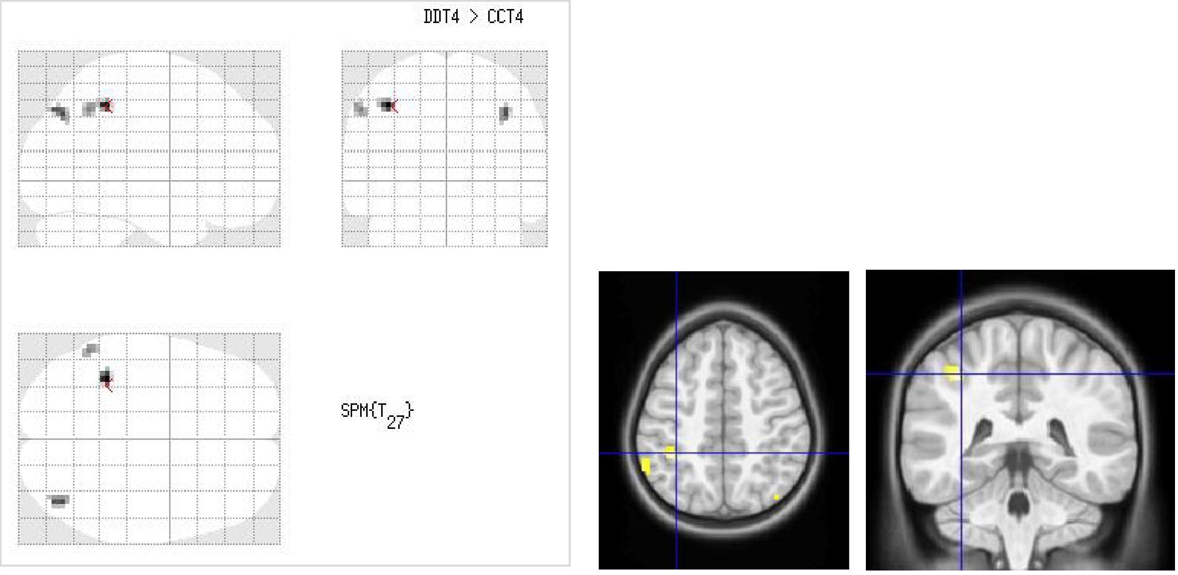

Figure S3. fMRI group differences at follow-up. Activation for the contrast DD vs. TD children at follow-up depicted on a glass brain (left side) and section (z = 48, y = -42) of a pediatric template (Fonov et al., 2011, 2009) ($p < .001$, $k \geq 19$, cluster-extent corrected).

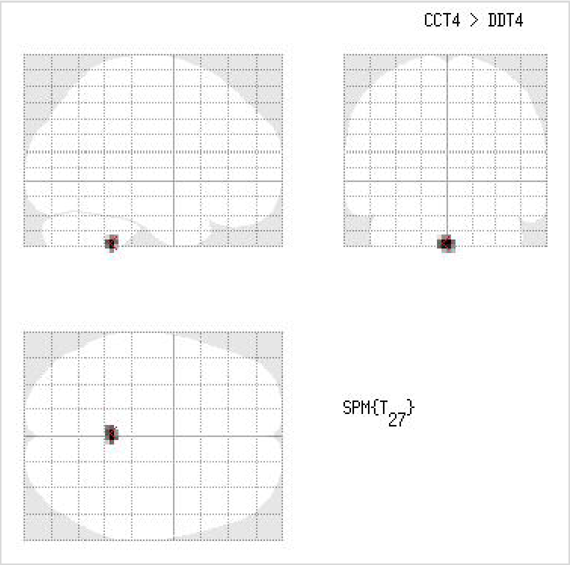

Figure S4. fMRI group differences at follow-up. Activation for the contrast TD vs. DD children at follow-up depicted on a glass brain ($p < .001$, $k \geq 19$, cluster-extent corrected).

4.2 fMRI developmental effects

Developmental changes took place in the DD group, showing increased activation in the right inferior parietal lobe (IPL) (supramarginal gyrus (SMG)) (x = 43, y = -35, z = 42), right angular gyrus (AG) (x = 37, y = -68, z = 45), and left superior parietal lobe (SPL) (x = -17, y = -47, z = 63). In the frontal lobe increased activation was found in the right middle frontal gyrus (MFG) (x = 37, y = 19, z = 48), right caudate nucleus (x = 22, y = 19, z = 12) as well as in the left insula (x = -26, y = 22, z = -6), left inferior (IFG) (x = -44, y = 37, z = 0) and superior frontal gyrus (SFG) (x = -8, y = 13, z = 45), left parahippocampal gyrus (PHG) (x = -26, y = -14, z = -27) and left thalamus (x = -14, y = -14, z = -3). DD children showed also an increase in activation in the right cerebellum (x = 10, y = -83, z = -18), left temporal pole (x = -50, y = 10, z = -21) and left middle temporal gyrus (MTG) (x = -50, y = -38, z = -3) (all activation at a threshold of $p < .005$) (Figure S5, upper left side and bottom). No decrease in activation was found in the DD group over development (Figure S5, upper right side).

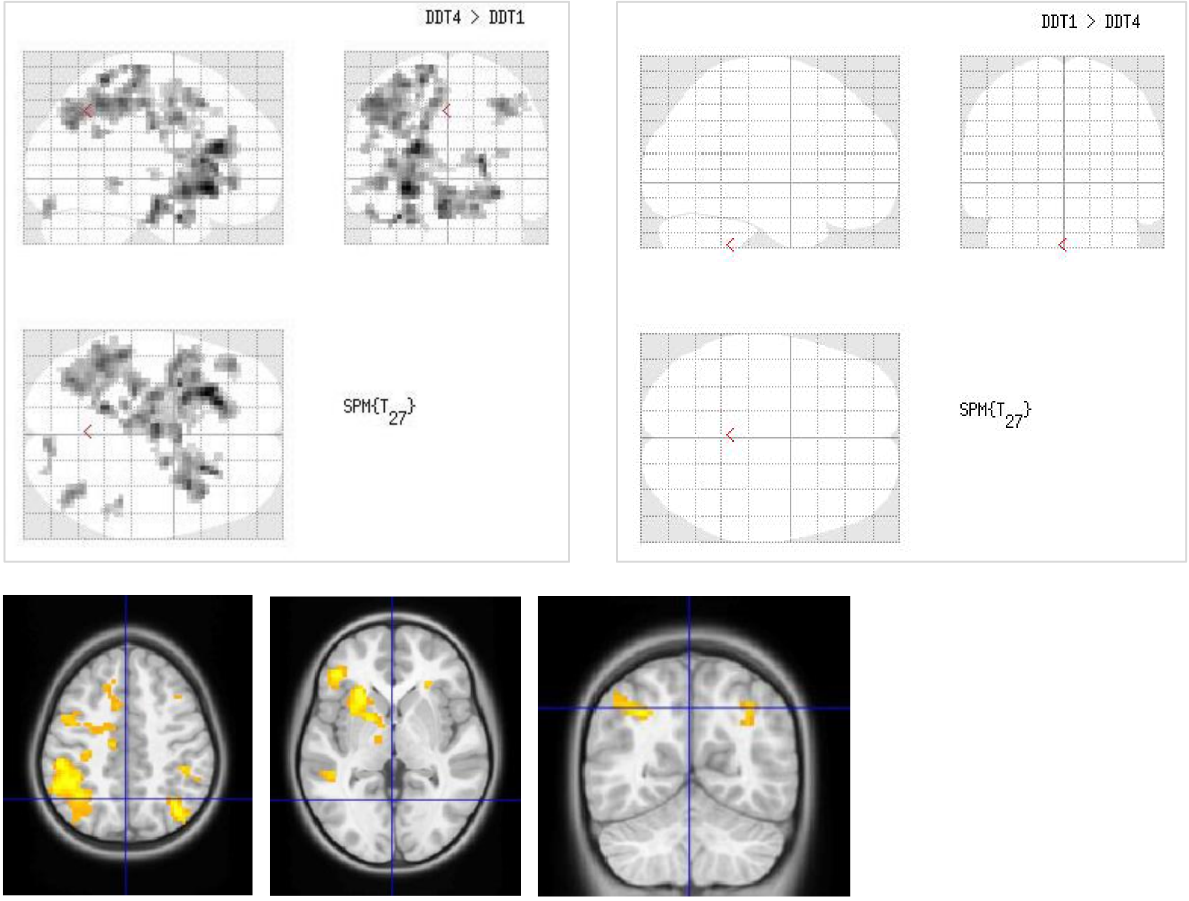

Figure S5. fMRI developmental effects in children with developmental dyscalculia. Upper left side and bottom. Activation increase over development for DD children depicted on a glass brain and section (z = 44, z = 0, y = -58) of a paediatric template (Fonov et al., 2011, 2009). Upper right side. No activation decrease was found in DD children over development ($p < .005$, $k \geq 22$, cluster-extent corrected).

TD children did not show any increase in activation over development at the statistical threshold of $p < .005$ (Figure S6, left side). An activation decrease was found in the right superior frontal gyrus (SFG) (x = 22, y = 40, z = 36) for the TD group (Figure S6, right side).

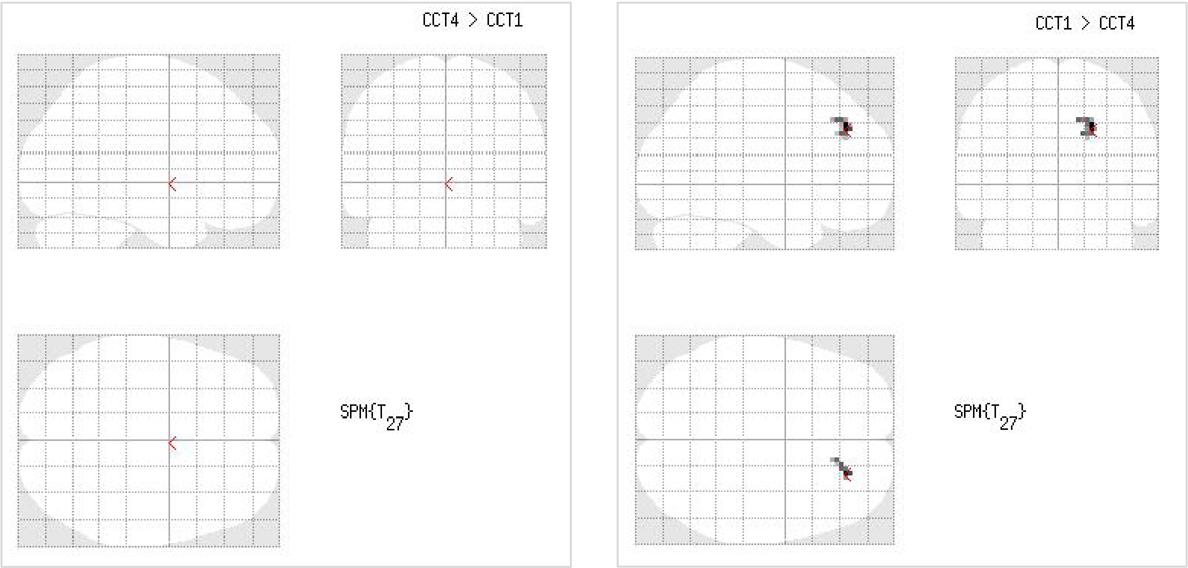

Figure S6. fMRI developmental effects in typically developing children. Left side. Activation increase over development for TD children depicted on a glass brain. Right side. Activation decrease over development in TD children ($p < .005$, $k \geq 22$, cluster-extent corrected).
